# Genetic background modifies host and viral parameters of neonatal gammaherpesvirus infection

**DOI:** 10.1128/jvi.00650-26

**Published:** 2026-08-14

**Authors:** M. A. Brown, E. R. Johansen, X. G. Bradeen, C. R. Rahlf, E. V. Xie, V. L. Tarakanova

**Affiliations:** 1 Department of Microbiology and Immunology, Medical College of Wisconsin 5506, Milwaukee, Wisconsin, USA; 2 Medical Scientist Training Program, Medical College of Wisconsin 5506, Milwaukee, Wisconsin, USA; 3 Cancer Center, Medical College of Wisconsin 5506, Milwaukee, Wisconsin, USA; Dartmouth College Geisel School of Medicine, Hanover, New Hampshire, USA

**Keywords:** B cell, chronic infection, genetic background, neonatal infection, gammaherpesvirus

## Abstract

Gammaherpesviruses are ubiquitous pathogens that establish lifelong infection and are associated with cancer and multiple sclerosis. Primary infection during adolescence significantly increases the risk of gammaherpesvirus-driven disease in humans. Correspondingly, infection of adolescent mice with murine gammaherpesvirus 68 (MHV68) has served as a tractable animal model to define host factors that control chronic gammaherpesvirus infection and pathogenesis. However, a majority of humans are infected with gammaherpesviruses during early life when the nature of adaptive immune responses is altered as compared to that of older children and adults. Given that human and murine gammaherpesviruses manipulate B cell differentiation to establish the latent reservoir in adolescents, this study aims to define the host and viral parameters of chronic gammaherpesvirus infection following neonatal inoculation. Similar to adolescents, MHV68-driven germinal center responses and the establishment of a latent viral reservoir in splenic germinal center B cells of BALB/c mice were reliably observed following neonatal intranasal infection with 400 PFU of MHV68. Unexpectedly, MHV68-driven splenomegaly, a ubiquitous clinical manifestation of gammaherpesvirus infection in recently infected adolescent mice and humans, was only observed in neonatally infected BALB/c males and not females. Interestingly, C57BL/6J neonates were largely resistant to the establishment of latent splenic infection following 400 PFU intranasal inoculation. Increasing the MHV68 inoculum rescued the establishment of chronic splenic infection and MHV68-driven germinal center responses in neonatally infected C57BL/6J mice. Thus, the current study unveils the role of genetic background and early life exposure in the susceptibility to systemic gammaherpesvirus infection.

**IMPORTANCE:**

Primary gammaherpesvirus infection of adolescents is associated with increased risk of gammaherpesvirus-driven disease, such as cancer and multiple sclerosis. Therefore, the murine gammaherpesvirus 68 (MHV68) animal model has served as a tractable experimental system to define host factors involved in control of gammaherpesvirus infection and pathogenesis following adolescent infection. However, a majority of human gammaherpesvirus infections occur during early childhood, a stage of human development that is marked by unique differences in the adaptive immune responses as compared to that of adults. This study demonstrates that the genetic background plays an important role in limiting systemic gammaherpesvirus infection following neonatal viral exposure, in contrast to that historically observed in primary adolescent infection. However, when susceptible to systemic infection, the extent of MHV68-driven germinal center responses and establishment of a latent viral reservoir in germinal center B cells are largely comparable between neonatally infected mice and published observations in adolescent MHV68 infection.

## INTRODUCTION

Gammaherpesviruses, such as human Epstein-Barr virus (EBV) and Kaposi’s sarcoma-associated herpesvirus (KSHV), establish lifelong infection in a majority of adults and are associated with cancer and multiple sclerosis (1, 2). Seroconversion at or past adolescence (ages 13–19) significantly increases the risk of EBV-driven malignancies and autoimmune disease as compared to childhood infection (age 12 or lower) (2–4). Primary EBV infection during adolescence, but not early childhood, is associated with a distinct clinical disease termed infectious mononucleosis (IM) (5). Clinically, IM presents with protracted splenomegaly and lymphadenopathy that is driven by an exaggerated activation of the host immune system. Interestingly, in addition to EBV-specific B and T cell responses, there is also a transient yet robust rise in activation and differentiation of B cells encoding B cell receptors that recognize self- and foreign species antigens. In fact, the Paul-Bunnell test that is clinically used to diagnose primary EBV infection in adolescents detects antibodies against sheep red blood cells and not EBV antigens (3, 6).

Primary EBV infection in prepubescent children is largely subclinical or entirely asymptomatic. The first prospective study of childhood EBV infection in a cohort of Minnesota children (18 months to 11.99 years of age) approximated the incidence of pediatric EBV infection at 9.2 cases/100 person-years with a high sibling concordance rate within the same family (4). Interestingly, in this pediatric cohort, approximately half of all primary pediatric EBV infections were symptomatic (4). In a retrospective study of the U.S. pediatric population, EBV seroprevalence remained at <50% in children younger than 11 years of age, with male children displaying lower EBV antibody titers than female children, independent of age (7). Despite the mostly subclinical nature of pediatric EBV infection in developed countries, it is associated with the development of specific cancers, especially in developing countries. For example, EBV infection in combination with *Plasmodium falciparum* infection is associated with endemic Burkitt’s lymphoma in pediatric populations in Sub-Saharan Africa (8). EBV is also associated with 20%–50% of Hodgkin’s lymphoma cases in the United States and Europe, with higher rates observed in developing countries (9). Hodgkin’s lymphoma in pediatric populations and in older adults tends to be EBV positive, whereas Hodgkin’s lymphoma cases in adolescents are more likely to be EBV negative (9, 10). Therefore, despite the apparent subclinical nature of primary infection, there remains a need to further define EBV pathogenesis in pediatric populations.

Given the species specificity of EBV and KSHV, infection with the genetically and biologically related murine gammaherpesvirus 68 (MHV68) has served as a powerful animal model to define host and viral factors that regulate chronic infection and viral pathogenesis (11). Due to the increased short- and long-term pathogenesis of adolescent EBV infection, an overwhelming majority of MHV68 studies model infection of adolescents. Similar to that observed for EBV, MHV68 infection of adolescent mice (aged 6–7 weeks old) presents with splenomegaly, lymphadenopathy, and robust nonspecific immune activation peaking at 16 days post-infection. As observed during IM, MHV68 infection of adolescent mice is associated with a robust yet transient rise in self- and foreign-antigen reactive class-switched antibodies, with several published studies defining host and viral factors that regulate MHV68-driven differentiation and infection of self-reactive B cells (12–15).

Importantly, >70% of humans acquire EBV infection prior to 15 years of age in North America (16), with >93% of EBV seroconversion observed in Uganda by 1 year of age (17). There are important differences between neonatal and adult immune responses that are likely to shape the outcome and pathogenesis of gammaherpesvirus infection. Both EBV and MHV68 infect naïve B cells and drive subsequent germinal center-based differentiation of infected and bystander B cells to support establishment of a latent viral reservoir in adolescent humans and mice (18–20). Neonatal B cells have decreased ability to undergo germinal center responses following immunization, requiring multiple immunogen exposures to induce germinal center responses (21). Furthermore, robust isotype class-switching observed in adolescent and adult germinal center responses is significantly attenuated in neonates, even when germinal center responses are induced (21). In contrast to attenuated germinal center responses and class-switching, neonatal CD8 T cells offer increased protection against influenza infection, largely due to their distinct lineage and innate-like, T cell receptor (TCR)-independent functions as compared to adult CD8 T cells (22, 23).

In addition to age, genetic background is an important modifier of immune responses and susceptibility to infection. C57BL/6J mice are more resistant to *Leishmania tropica* and *Chlamydia muridarum* infections compared to BALB/c mice (24, 25). The resistance of C57BL/6J mice to *Chlamydia muridarum* infection was associated with higher IFNγ levels in infected lungs and skewing of immune responses toward T helper type 1 vs T helper type 17 (24). *Mycobacterium tuberculosis*-infected C57/BL6J mice displayed increased IFNγ levels and decreased numbers of regulatory T cells in the lungs, as compared to infected BALB/c mice (26). Similar genetic background-driven skewing of the immune responses is observed following infection with *Leishmania major* (27–29).

Given the increased EBV pathogenesis following adolescent primary infection (as defined by the incidence of IM), a vast majority of publications in the MHV68 field define viral and host parameters of chronic gammaherpesvirus infection and pathogenesis following inoculation of adolescent mice. In contrast, only a few published studies describe host and viral parameters of neonatal MHV68 infection (30–33). A foundational 2008 publication from the Rochford group demonstrated similar MHV68 genome copies in lungs and mediastinal lymph nodes of acutely infected BALB/c neonates and adolescents at 6 days post-infection (32). MHV68 DNA was also detected in the heart, spleen, and liver of acutely infected neonates but not adolescents. Long-term MHV68 DNA loads (30 days post-infection) were similar in the lymph nodes and spleen of adolescently and neonatally infected BALB/c mice, with some MHV68 DNA detected in lungs and kidneys/adrenal glands following neonatal, but not adolescent primary infection (32). Finally, splenomegaly observed in MHV68-infected neonates at 16 days post-infection was significantly attenuated as compared to adolescent-infected mice (32). Importantly, neither the parameters of MHV68-driven B cell differentiation nor the infection of B cells was measured (32).

This study aims to define host and viral parameters of chronic MHV68 infection following neonatal inoculation. Following intranasal inoculation of BALB/c neonates with 400 PFU of MHV68, viral latent reservoir and reactivation in the spleen were reproducibly detected at 16 days post-infection, similar to that previously described for adolescently infected mice (34). Furthermore, MHV68 inoculation of neonatal BALB/c mice resulted in induction of similar germinal center responses in male and female littermates, with virus-induced splenomegaly only observed in male mice. Similar to adolescently infected mice, MHV68 latency was primarily established in splenic germinal center B cells in neonatally infected BALB/c mice. As expected and despite induction of the germinal center response, neonatally infected mice failed to effectively generate class-switched antibodies against MHV68 or self-antigens, as compared to that observed in adolescents. However, an increase in anti-MHV68 IgM titers was observed in neonatally infected BALB/c mice. When these studies were repeated in C57BL/6J mice, MHV68 failed to reproducibly establish a latent reservoir in the spleen following 400 PFU intranasal inoculation. A subsequent 10-fold increase in the viral inoculum largely recapitulated the viral and host parameters of MHV68 infection observed in BALB/c mice neonatally infected with a lower dose of MHV68. In summary, the current study defines host and viral parameters of neonatal MHV68 infection, refining an experimental model that can be harnessed to define gammaherpesvirus-host interactions in neonates.

## RESULTS

### MHV68 readily establishes latency in the spleens of BALB/c but not C57BL/6J neonatal mice

The timing and viral parameters of natural MHV68 infection are largely defined by numerous studies of adolescently infected mice and only a few published studies of neonatal MHV68 infection (30, 32, 33, 35). Following intranasal MHV68 inoculation, virus acutely replicates in the lungs, with infectious virus cleared ~12–15 days post-infection, regardless of the age at infection (32, 34). Concurrent with the clearance of infectious virions, there is a rise in the latent splenic reservoir, which peaks at 16 days post-adolescent infection (34). MHV68 DNA was detected in neonatally infected BALB/c mice as early as 6 days post-infection and persisted for at least 30 days post-infection (32). However, the published neonatal MHV68 infection study (32) exclusively assessed MHV68 genome copy numbers in the spleen, an approach that does not distinguish between the latent and lytic viral life cycle. To more precisely define viral parameters of splenic infection, BALB/c littermates were intranasally infected with 400 PFU of MHV68 at 8–10 days of life, and splenic MHV68 infection was assessed 16–17 days later, with the timing chosen based on the timing of peak splenic latency observed in adolescently infected mice.

**Fig 1:**
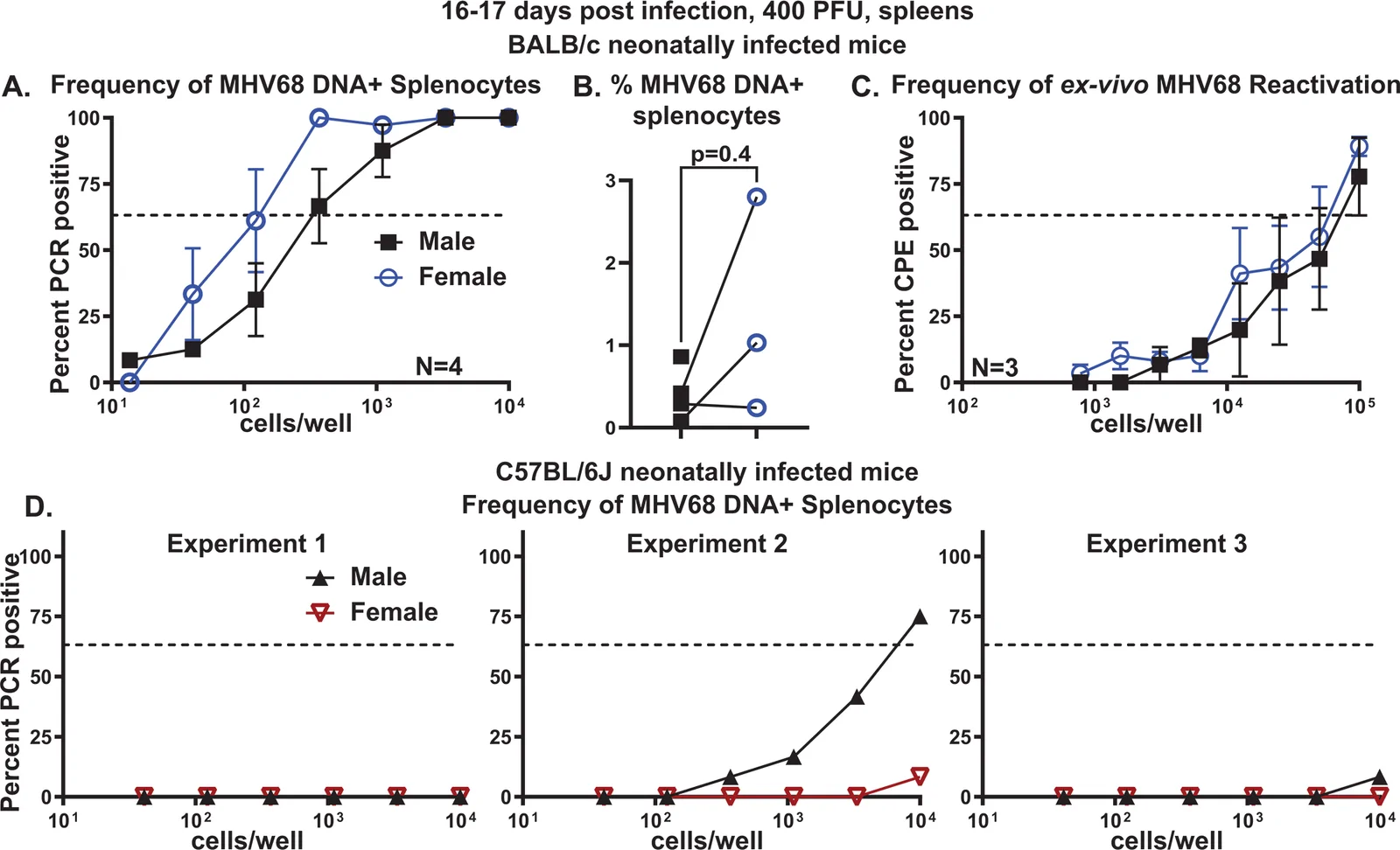
MHV68 readily establishes latency in the spleens of BALB/c but not C57BL/6J neonatal mice. BALB/c (**A through C**) and C57BL/6J (**D**) littermate mice between 8 and 10 days of age were intranasally inoculated with 400 PFU of MHV68. Male and female splenocytes were analyzed by limiting dilution assays at 16–17 days post-infection. (**A**) The frequency of MHV68 DNA-positive splenocytes in BALB/c neonatally infected mice was determined by limiting dilution PCR. In this and other limiting dilution studies presented in the manuscript, the intersection of the sigmoidal curve and the dotted line drawn at Y = 63.2% indicates the inverse frequency of positive events as defined by the corresponding x-coordinate. For all viral limiting dilution assays presented in this study, splenocytes from male or female mice in each litter were pooled separately, with limiting dilution assays performed on each pooled population. Data for panel A were compiled from four independent litters (*N* = 4), with distribution of mice as follows: experiment (Exp) 1: male (M) = 3, female (F) = 0; Exp 2: M = 3, F = 7; Exp 3: M = 2, F = 4; Exp 4: M = 4, F = 3. (**B**) The inverse frequency of MHV68+ splenocytes was converted to %MHV68 DNA-positive cells, displayed for each independent study. Connected symbols represent data from the same study. (**C**) The frequency of *ex vivo* MHV68 reactivation from splenocytes of neonatally infected BALB/c mice was determined by limiting dilution assay (LDA). Data for panel C were compiled from three independent litters (*N* = 3), with distribution of mice as follows: Exp 1: M = 3, F = 2; Exp 2: M = 3, F = 7; Exp 3: M = 2, F = 4. (**A and C**) Mean and standard error of the mean are shown. (**D**) The frequency of MHV68 DNA-positive splenocytes in neonatally infected C57BL/6J mice was determined by limiting dilution PCR. Data from three independent litters are shown, with distribution of mice as follows: Exp 1: M = 3, F = 3; Exp 2: M = 5, F = 5; Exp 3: M = 4, F = 4.

Sex differences in the clinical presentation of EBV-driven infectious mononucleosis have been reported in adolescents and adults, including increased leukocyte counts in the males and increased levels of liver transaminases in females (36). EBV-associated multiple sclerosis is more prevalent in females, whereas childhood Hodgkin’s lymphoma, frequently associated with EBV infection, has a high male bias (37). Similarly, males are more likely to shed KSHV in saliva compared to females (38), and the incidence of Kaposi’s sarcoma has a strong male bias (39). In contrast, adolescently infected C57BL/6J male and female mice display similar viral and host parameters of chronic MHV68 infection in the spleen ( E. R. Johansen, X. G. Bradeen, E. Xie, C. R. Rahlf, and V. L. Tarakanova, unpublished data). Because neonatal infection occurs in a highly controlled environment that allows pups of both sexes to share the same cage and food source, MHV68 infection was assessed in male and female mice within each litter to determine any sex-dependent differences. MHV68 DNA+ splenocytes were reliably detected in the spleens of BALB/c mice at 16–17 days after neonatal infection (Fig. 1A and B). Frequencies of *ex vivo* MHV68 reactivation from splenocytes were similar in neonatally infected males and females at 16–17 days post-infection, with no persistent replication detected (Fig. 1C; data not shown). Thus, neonatal MHV68 infection of BALB/c mice resulted in reliable establishment of a splenic latent reservoir and viral reactivation following a 400 PFU intranasal dose.

Most published MHV68 studies use adolescent mice with the C57BL/6J genetic background; these animals uniformly establish a latent splenic reservoir even after a very low-dose intranasal inoculation (40 PFU) (34). While direct comparison of the MHV68 parameters between adolescently infected C57BL/6J and BALB/c mice has not been published, adolescently infected BALB/c mice also readily support the establishment of chronic infection (40). To determine the extent to which genetic background modifies chronic MHV68 infection of neonates, 8–10 days old C57BL/6J littermates were intranasally inoculated with 400 PFU of MHV68, and parameters of splenic infection were measured 16–17 days later. Surprisingly, and in contrast to BALB/c neonates, C57BL/6J neonates were more resistant to the establishment of chronic MHV68 infection in the spleen. Very few, if any, MHV68 DNA+ splenocytes were detected in two of three independent studies at 16–17 days post-infection (experiments 1 and 3, Fig. 1D), with one of three studies demonstrating a low frequency of MHV68 DNA+ splenocytes in males, with a further decrease in female splenocytes (Fig. 1C, experiment 2). Thus, genetic background defined the susceptibility of neonates to the establishment of chronic gammaherpesvirus infection in the spleen following low-dose intranasal infection, with potential reasons highlighted later in the text.

### MHV68 infection of BALB/c neonates drives an increase in germinal center response

MHV68 infection of adolescent mice is associated with splenomegaly and a robust germinal center response. The germinal center response is critical for the establishment of latent MHV68 infection in adolescent spleens, as the majority of latently infected splenocytes are represented by germinal center B cells at 16 days post-adolescent infection (20). CD4 T follicular helper cells that localize to the germinal centers, while not infected, are critical to support proliferation and differentiation of germinal center B cells and the MHV68 latent reservoir in the spleen (41, 42).

**Fig 2:**
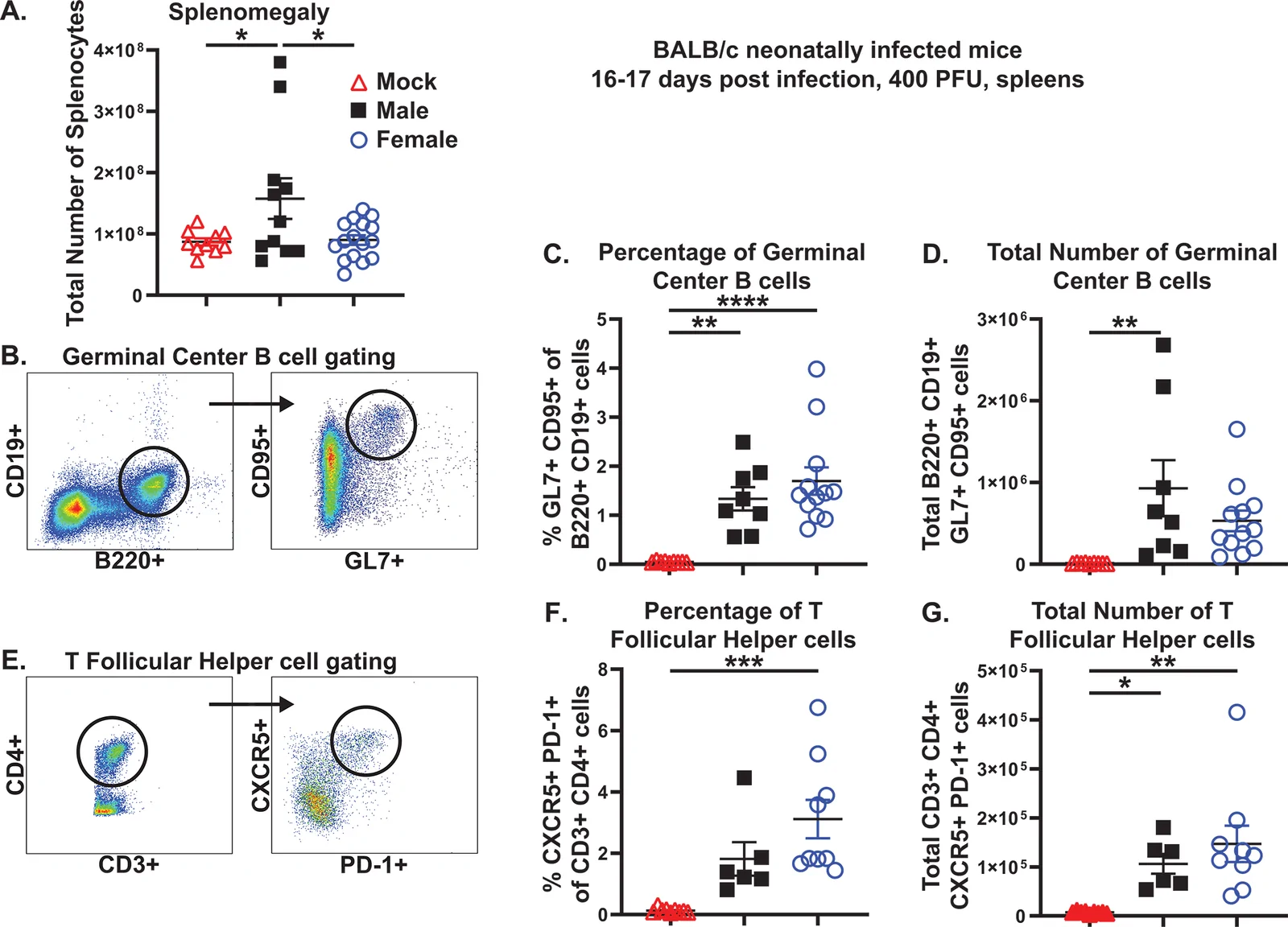
MHV68 infection of BALB/c neonates drives an increase in germinal center response. BALB/c litters were inoculated with MHV68 as in Fig. 1, and splenocytes from individual mice were analyzed at 16–17 days post-infection. Age-matched uninfected litters were used as mocks, with data from males and females pooled within the mock group. (**A**) The total number of splenocytes per mouse. Data for panel A were compiled from four independent litters (*N* = 4), with distribution of mice as follows: Exp 1: M = 3, F = 2; Exp 2: M = 3, F = 7; Exp 3: M = 2, F = 3; Exp 4: M = 3, F = 4. A fifth litter with only mock-infected mice was also included in the analysis, M = 6, F = 3. Total numbers of mice: mock = 9, male = 11, female = 16. (**B**) Flow cytometry gating strategy for germinal center B cells defined as CD19^+^B220^+^GL7^+^CD95^+^. The frequency (**C**) and absolute number (**D**) of germinal center B cells. Data for panels C and D were compiled from three independent litters (*N* = 3), with distribution of mice as follows: Exp 1: M = 3, F = 2; Exp 2: M = 3, F = 7; Exp 3: M = 2, F = 3; a fourth litter with only mock-infected mice was also included in the analysis, M = 6, F = 3. Total numbers of mice: mock = 9, male = 8, female = 12. (**E**) Flow cytometry gating strategy for T follicular helper cells defined as CD3^+^CD4^+^CXCR5^+^PD-1^+^. The frequency (**F**) and absolute number (**G**) of T follicular helper cells. Data for panels F and G were compiled from two independent litters (*N* = 2), with distribution of mice as follows: Exp 1: M = 3, F = 2; Exp 2: M = 3, F = 7; a third litter with only mock-infected mice was also included in the analysis, M = 6, F = 3. Total numbers of mice: mock = 9, male = 6, female = 9. Each symbol represents an individual spleen; mean and standard error of the mean are shown. *P < 0.05; **P < 0.01; ***P < 0.001, ****P < 0.0001.

Given the reported attenuation of splenomegaly observed in neonatally infected as compared to adolescently infected BALB/c mice (32), splenomegaly was assessed in male and female BALB/c littermates at 16–17 days post-infection. An increase in the total number of splenocytes was observed in infected BALB/c males as compared to uninfected age-matched controls at 16–17 days post-infection (Fig. 2A). The observed increase in splenic cellularity was comparable in magnitude to the increase in infected neonatal spleen weight in the published study (32). Surprisingly, the total number of splenocytes was not increased above baseline following neonatal MHV68 infection of BALB/c females (Fig. 2A).

Given the importance of the germinal center response for the establishment of the splenic MHV68 latent reservoir in adolescents, germinal center B cells and T follicular helper cells were assessed next. Proportion and number of germinal center B cells were increased in neonatally infected BALB/c female and male spleens at 16–17 days post-infection, as compared to age-matched uninfected mice, with no sex-dependent differences observed (Fig. 2C and D, gating strategy in Fig. 2B). Similarly, MHV68 infection was associated with increased proportion and frequency of T follicular helper cells with no sex-dependent phenotypes (Fig. 2F and G, gating strategy in Fig. 2E). In summary, establishment of latent MHV68 infection in the spleen coincided with increased germinal center responses in neonatally infected BALB/c males and females, with increased splenocyte numbers only observed in neonatally infected males.

### MHV68 establishes latency in germinal center B cells in neonatal BALB/c mice

**Fig 3:**
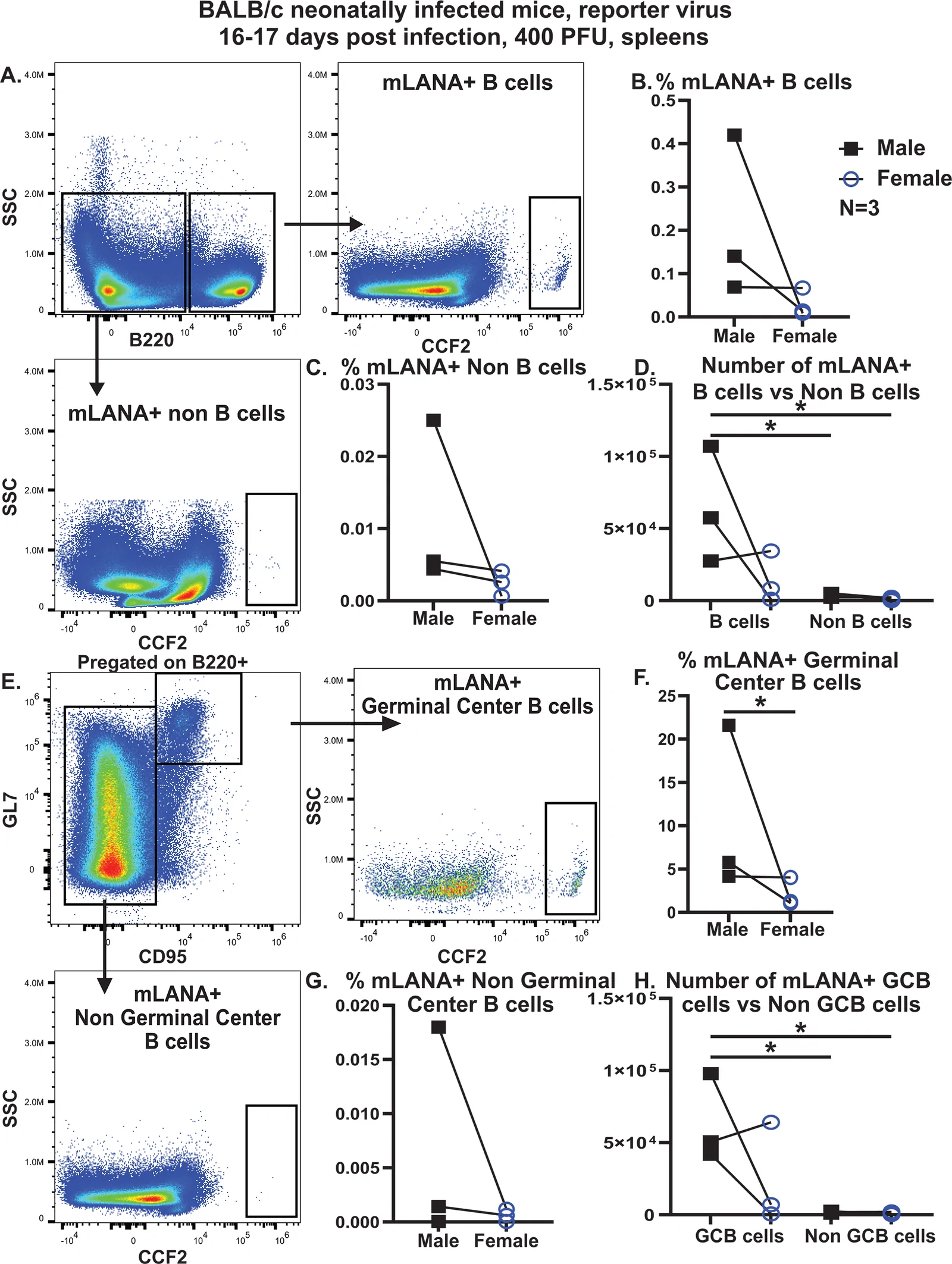
MHV68 establishes latency in germinal center B cells in neonatal BALB/c mice. BALB/c litters between 8 and 10 days of age were intranasally inoculated with 400 PFU MHV68.orf73βla. Male and female splenocytes were pooled separately within each litter and analyzed by flow cytometry at 16–17 days post-infection. (**A**) Gating strategy for identification of total B cells (B220^+^) and non-B cells (B220^−^) expressing mLANA-β-lactamase fusion protein. The frequency of mLANA+ B cells (**B**) and non-B cells (**C**). (**D**) The absolute number of mLANA+ B cells and non-B cells. (**E**) Gating strategy for identification of total germinal center B cells (CD19^+^B220^+^GL7^+^CD95^+^) and non-germinal center B cells expressing mLANA-β-lactamase fusion protein. The frequency of mLANA+ germinal center B cells (**F**) and non-germinal center B cells (**G**). (**H**) The absolute number of mLANA+ germinal center B cells and non-germinal center B cells. (**B through D and F through H**) Data were compiled from three independent litters, with distribution of mice as follows: Exp 1: M = 4, F = 3; Exp 2: M = 1, F = 5; Exp 3: M = 3, F = 5. Each symbol represents pooled splenocytes of males or females within an independent experiment. Observations from the same experiment are connected by a line. *P < 0.05.

EBV and MHV68 manipulate B cell differentiation to establish chronic infection in adolescents (19, 20). Specifically, these gammaherpesviruses drive infected naïve B cells through the germinal center response, where the latent reservoir is amplified by cellular proliferation. Subsequent differentiation of latently infected germinal center B cells into plasma cells supports MHV68 and EBV reactivation (43–45). To define the extent to which splenic B cells supported MHV68 latent reservoir, BALB/c neonates were infected with the MHV68.orf73βla reporter virus. MHV68.orf73βla expresses a fusion between *orf73*-encoded mLANA, a critical MHV68 latent protein, and β-lactamase (46), enabling the detection of infected cells by flow cytometry using a cell-permeable β-lactamase substrate. Similar to that observed following adolescent infection, the highest frequency of infected splenocytes was observed in splenic B cells, as compared to B220-negative splenocytes of BALB/c mice infected neonatally (Fig. 3B and C, gating strategy in A). The frequency of infected B cells was not statistically different between BALB/c males and females (Fig. 3B, *P* = 0.1). When the frequency of infection was converted to absolute numbers of infected cells, splenic B cells were found to support a vast majority of the latent MHV68 reservoir in neonatally infected BALB/c mice as compared to non-B cells (Fig. 3D).

Next, infection of germinal center B cells versus other splenic B cells was assessed (using gating strategy in Fig. 3E). As in adolescents, a high frequency of germinal center B cells was MHV68+ in neonatally infected BALB/c mice, with the frequency of infection higher in males as compared to females (Fig. 3F). The frequency of infected germinal center B cells was greater than the frequency of infected non-germinal center B cells (Fig. 3F and G). When the frequency of infection was converted to the absolute number of infected cells, splenic germinal center B cells were found to support a majority of the latent MHV68 reservoir in neonatally infected BALB/c mice compared to non-germinal center B cells (Fig. 3H). In summary, MHV68 preferentially established a latent splenic reservoir in germinal center B cells of BALB/c mice following neonatal infection, similar to that observed following adolescent MHV68 infection.

### MHV68 infection of BALB/c neonates induces minimal class-switched humoral responses

**Fig 4:**
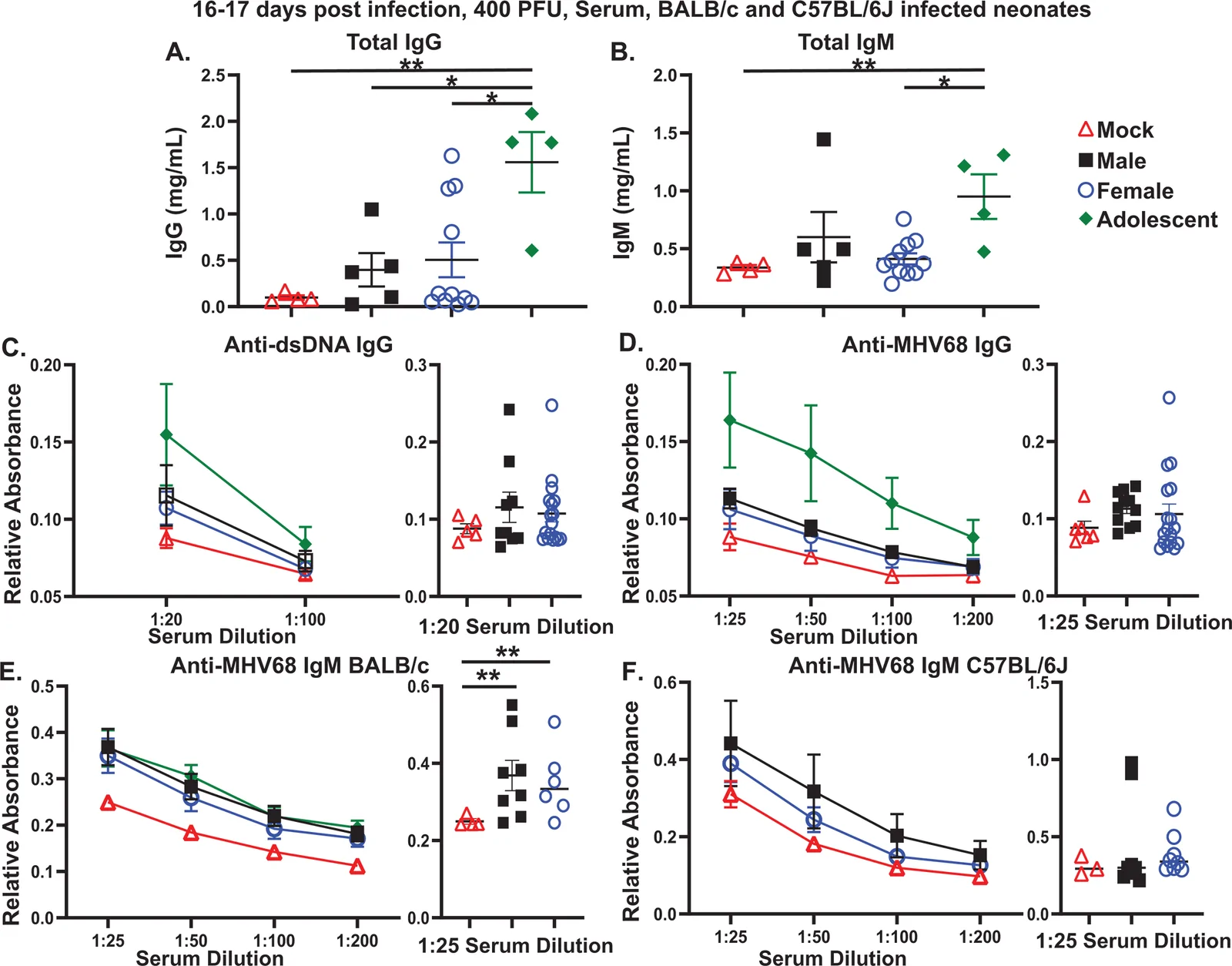
MHV68 infection of BALB/c neonates induces minimal class-switched humoral responses. BALB/c (**A through E**) and C57BL/6J (**F**) littermates were infected with WT MHV68 as in Fig. 1. Serum from individual mice was analyzed by enzyme-linked immunosorbent assay (ELISA) at 16–17 days post-infection. Total serum IgG (**A**) and IgM (**B**) titers. Each symbol represents an individual animal. Data for panels A and B were compiled from two independent litters (*N* = 2), with distribution of mice as follows: Exp 1: M = 3, F = 7; Exp 2: M = 2, F = 4. Total numbers of mice: mock = 4, male = 5, female = 11, adolescent = 4. For anti-double stranded DNA (dsDNA) IgG (**C**), anti-MHV68 IgG (**D**), and anti-MHV68 IgM from BALB/c (**E**) and C57BL/6J (**F**) neonates, the left panel represents pooled data at each dilution, while the right panel represents each individual mouse at the highest dilution. Data for panel C was compiled from four independent litters (*N* = 4), with distribution of mice as follows: Exp 1: M = 1, F = 2; Exp 2: M = 3, F = 7; Exp 3: M = 2, F = 4; Exp 4: M = 3, F = 4. Total numbers of mice: mock = 5, male = 9, female = 17, adolescent = 4. Data for panel D was compiled from four independent litters (*N* = 4), with distribution of mice as follows: Exp 1: M = 3, F = 2; Exp 2: M = 3, F = 7; Exp 3: M = 2, F = 4; Exp 4: M = 3, F = 4. Total numbers of mice: mock = 6, male = 11, female = 17, adolescent = 4. Data for panel E was compiled from two independent litters (*N* = 2), with distribution of mice as follows: Exp 1: M = 4, F = 3; Exp 2: M = 4, F = 3. Total numbers of mice: mock = 4, male = 8, female = 6, adolescent = 4. Data for panel F was compiled from two independent litters (*N* = 2), with distribution of mice as follows: Exp 1: M = 3, F = 3; Exp 2: M = 5, F = 5. Total numbers of mice: mock = 3, male = 8, female = 8, adolescent = 4. *P < 0.05, **P < 0.01.

Germinal center B cell differentiation is associated with isotype class switching and somatic hypermutation, both processes uniquely dependent on the expression of activation-induced cytidine deaminase. Differentiation of class-switched germinal center B cells into antibody-producing plasma cells results in increased IgG serum titers. Neonates have impaired germinal center responses and isotype class-switching, an impairment that can be partially overcome by sequential antigen exposures (21, 47). Having observed increased germinal center responses in neonatally infected BALB/c mice (Fig. 2), humoral responses were assessed next. While some heterogeneity was observed, no statistically significant difference in total IgG titers was found between age-matched uninfected controls (mock) and neonatally infected male or female BALB/c mice (Fig. 4A, serum from adolescently infected BALB/c mice at 16–17 days post-MHV68 infection was used as a positive control in this and subsequent panels). Total IgM serum titers also remained at baseline in males and females at 16–17 days post-neonatal infection (Fig. 4B).

A robust but transient induction in self- and foreign-antigen reactive antibodies is a hallmark of adolescent EBV and MHV68 infection (6), with the peak titers of self-reactive IgG observed at 16 days post-MHV68 infection (12, 13, 15). In contrast to that observed during adolescent infection, MHV68 infection of male and female BALB/c neonates failed to significantly increase IgG titers against double-stranded DNA, a representative self-reactive antibody, at 16–17 days post-infection (Fig. 4C). MHV68 infection of BALB/c male and female neonates also failed to increase anti-MHV68 IgG titers at 16–17 days post-infection in a majority of infected animals (Fig. 4D). Since neonates have impaired germinal center responses and isotype class-switching, anti-MHV68 IgM titers were assessed next in neonatally infected BALB/c mice. In contrast to that observed for IgG titers, MHV68 infection of neonatal BALB/c mice resulted in increased anti-MHV68 IgM titers in males and females (Fig. 4E). In summary, MHV68 infection of BALB/c neonates only induced an increase in anti-MHV68 IgM and was not sufficient to induce robust class switching to IgG at 16 days post-infection.

Having failed to reproducibly detect systemic MHV68 infection following 400 PFU inoculation of C57BL/6J neonates (Fig. 1D), seroconversion was tested next to determine if low-dose MHV68 inoculation generated any anti-MHV68 responses in C57BL/6J neonates. Specifically, serum anti-MHV68 IgM titers were measured in neonatally infected C57BL/6J mice from experiments 1 and 2 (as shown in Fig. 1D). Of all tested neonatally infected C57BL/6J mice, only two males and one female had elevated anti-MHV68 IgM titers compared to mock-infected controls following infection with 400 PFU of MHV68 (Fig. 4F). These findings suggest that 400 PFU inoculation of C57BL/6J neonates does not reliably induce seroconversion.

### Neonatal C57BL/6J mice do not have increased baseline interferon signaling in the lungs

**Fig 5:**
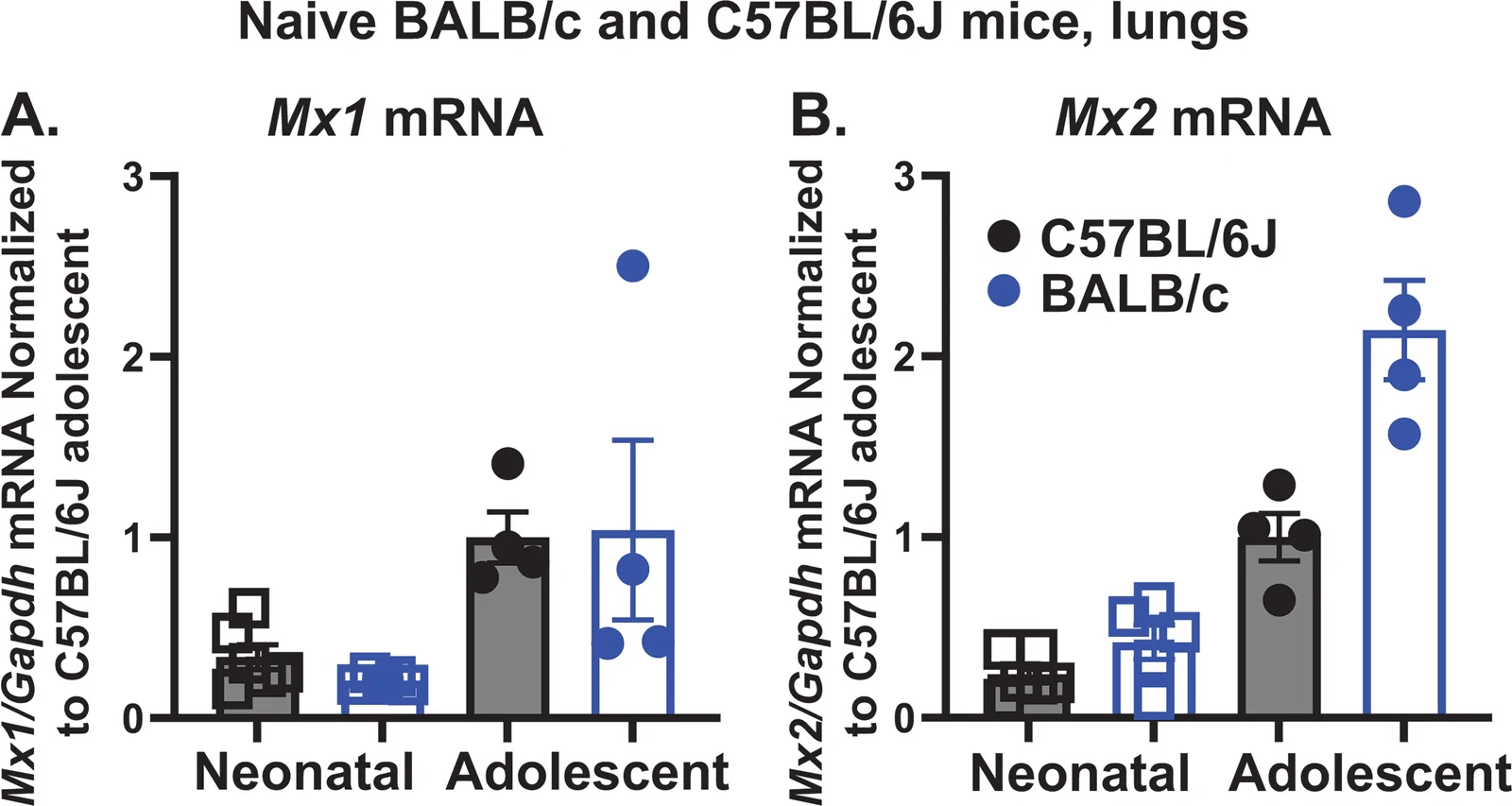
Neonatal C57BL/6J mice do not have increased baseline interferon signaling in the lungs. Total RNA was isolated from lungs of naïve BALB/c and C57BL/6J mice at 8–10 days of age (neonatal) or 6–7 weeks of age (adolescent). Total RNA was subjected to quantitative reverse transcription-PCR (qRT-PCR); each symbol represents an individual mouse. Data from females and males were pooled. Numbers of mice: neonatal C57BL/6J = 6, neonatal BALB/c = 5, adolescent C57BL/6J = 4, adolescent BALB/c = 4. Relative *Mx1* (**A**) and *Mx2* (**B**) mRNA levels normalized to *Gapdh* expression in each sample.

Intranasal low-dose MHV68 inoculation failed to reliably establish systemic MHV68 infection in the spleens of neonatally infected C57BL/6J mice (Fig. 1D). In contrast, the same conditions of infection led to a reliable and robust establishment of a latent MHV68 splenic reservoir in neonatally infected BALB/c mice. A recent study from the Nice group showed that increased baseline interferon (IFN) signaling in intestinal epithelial cells of neonatal mice conferred increased protection from rotavirus infection (48). Therefore, we hypothesized that C57BL/6J neonatal mice were more resistant to MHV68 infection due to higher baseline levels of IFN signaling in the lungs. To test this hypothesis, baseline IFN signaling was assessed by measuring mRNA levels of *Mx1* and *Mx2*, two representative IFN-stimulated genes (ISGs). Similar relative mRNA levels of *Mx1* and *Mx2* were observed in BALB/c and C57BL/6J lungs from uninfected neonates (8–10 days of age, Fig. 5A and B). Baseline *Mx1* and *Mx2* expression in lungs increased between neonatal and adolescent life stages in both genetic backgrounds (adolescent lungs obtained from uninfected 6–7 weeks old mice, Fig. 5A and B). In summary, similar levels of baseline IFN signaling in neonatal lungs of BALB/c and C57BL/6J mice failed to explain the resistance to low-dose intranasal MHV68 infection observed in C57BL/6J neonates.

### Analyses of persistent MHV68 replication and relative MHV68 DNA levels in lungs following neonatal infection

**Fig 6:**
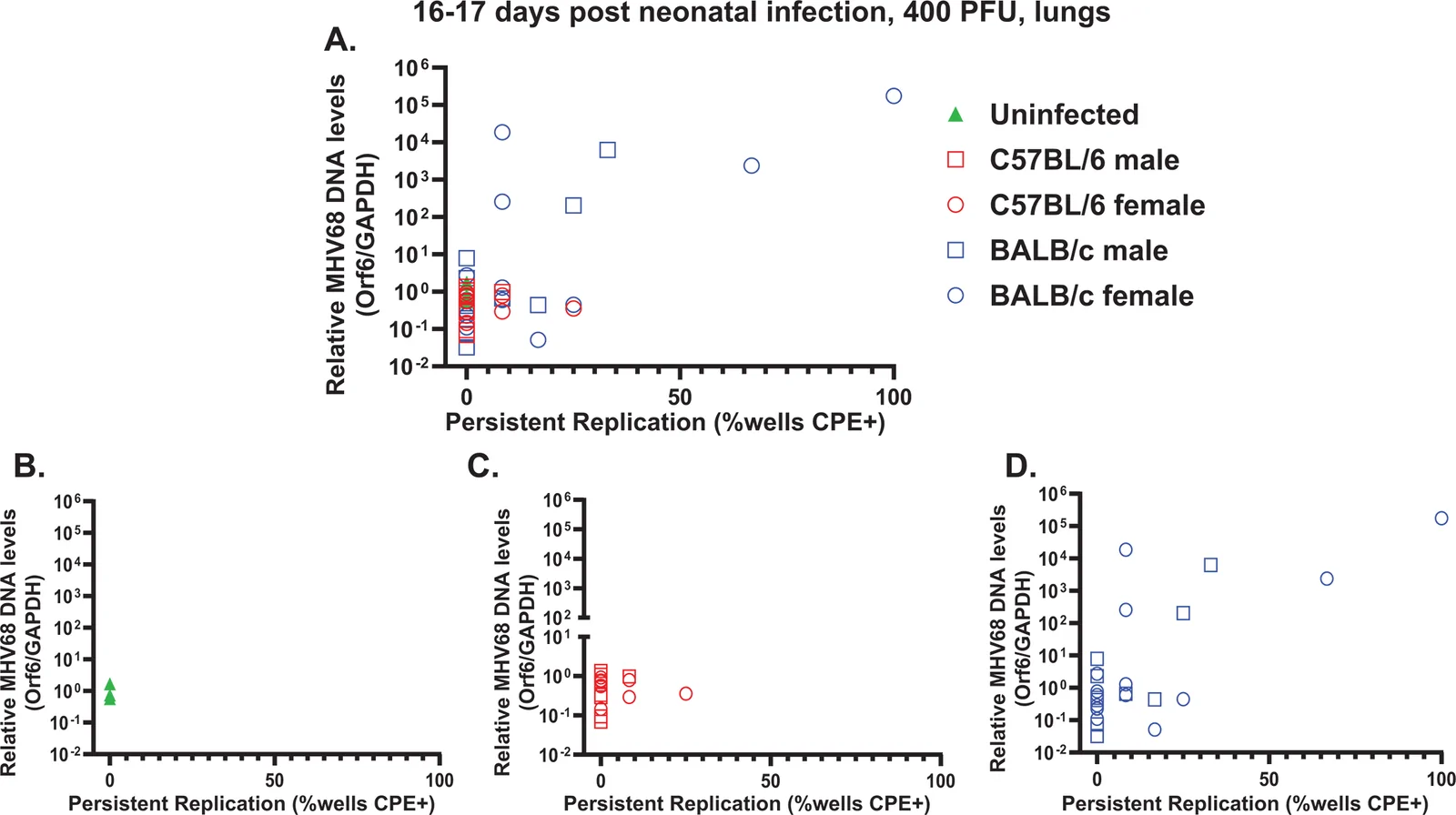
Analyses of persistent MHV68 replication and relative MHV68 DNA levels in lungs following neonatal infection. BALB/c and C57BL/6J littermates were infected with WT MHV68 as in Fig. 1. At 16–17 days post-infection, individual lungs were assessed for persistent MHV68 replication using a semi-quantitative highly sensitive limiting dilution assay and relative MHV68 DNA levels using quantitative PCR. (**A**) Combined plot showing persistent viral replication versus relative MHV68 DNA levels. Data in panel A were separated into subsequent individual plots for uninfected controls (*N* = 3) (**B**), C57BL/6J male (*N* = 8) and female (*N* = 10) infected neonates (**C**), and BALB/c male (*N* = 10) and female (*N* = 15) infected neonates (**D**). Each symbol represents an individual animal. Data for panel C were compiled from three independent litters (*N* = 3), with distribution of mice as follows: Exp 1: M = 2, F = 3; Exp 2: M = 3, F = 5; Exp 3: M = 3, F = 2. Data for panel D were compiled from four independent litters (*N* = 4), with distribution of mice as follows: Exp 1: M = 2, F = 2; Exp 2: M = 3, F = 6; Exp 3: M = 2, F = 3; Exp 4: M = 3, F = 4.

Neonatally infected BALB/c mice were reported to clear infectious virions from the lungs by 15 days post-infection (32), as measured using a mouse embryonic fibroblast (MEF)-based plaque assay. However, MHV68 DNA was detected in the BALB/c lungs as late as 30 days post-neonatal infection. Having observed resistance of C57BL/6J mice to the establishment of chronic splenic MHV68 reservoir following low-dose neonatal infection, the status of lung MHV68 infection was compared at 16–17 days following low-dose neonatal inoculation. Individual lungs were homogenized and subjected to parallel assessment of persistent MHV68 replication and relative MHV68 DNA levels, with results summarized in Fig. 6A. As expected, no persistent MHV68 replication was detected in uninfected lungs, with the relative MHV68 DNA levels set to an average of 1 in uninfected samples to define the limit of detection (Fig. 6B). Relative lung MHV68 DNA levels remained at or below the limit of detection in all examined C57BL/6J mice at 16–17 days post-neonatal infection with 400 PFU of MHV68 (Fig. 6C, 10 females and 8 males). However, despite the inability to detect MHV68 DNA, low levels of persistent MHV68 replication were detected in 4 C57BL/6J lungs (Fig. 6C, 3 females and 1 male). Most neonatally infected BALB/c mice displayed similar viral phenotypes, with MHV68 DNA levels at or below the limit of detection and few lungs demonstrating low levels of persistent viral replication (Fig. 6D, 15 females and 10 males). High MHV68 DNA levels detected in 4 female and 2 male BALB/c lungs did not uniformly translate into high levels of persistent replication (Fig. 6D). In summary, the relative MHV68 DNA levels were largely not predictive of the amount of persistent MHV68 replication in C57BL/6J and BALB/c lungs at 16–17 days following low-dose neonatal infection.

### Increased MHV68 inoculation dose leads to the establishment of splenic latency and MHV68-driven germinal center responses in C57BL/6J neonatal mice

**Fig 7:**
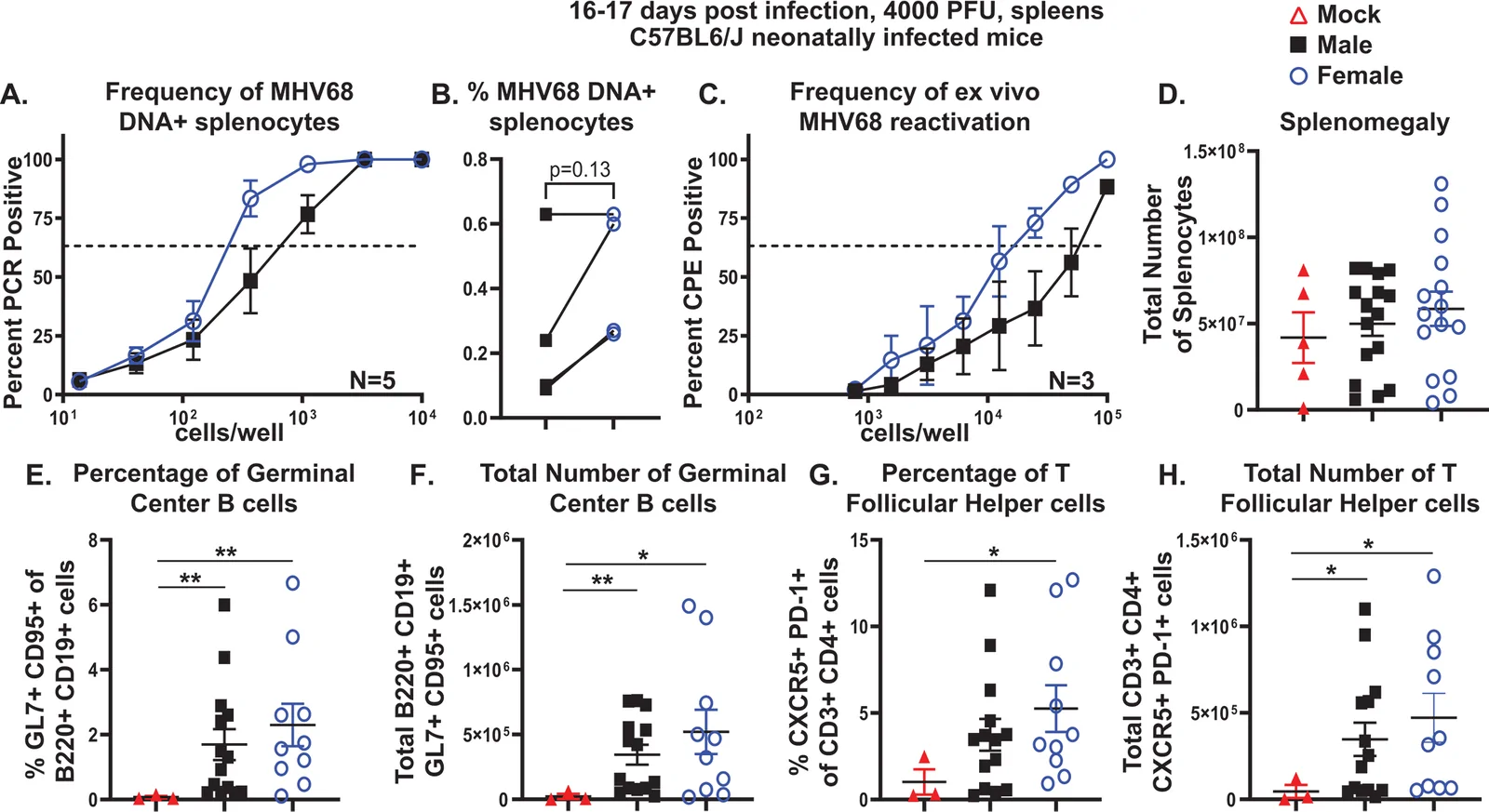
Increased MHV68 inoculation dose leads to the establishment of splenic latency and MHV68-driven germinal center responses in C57BL/6J neonatal mice. Male and female C57BL/6J littermates were intranasally inoculated with 4,000 PFU MHV68 at 8–10 days of age. Pooled male and female splenocytes (**A through C**) or splenocytes from individual mice in each experiment (**D through H**) were analyzed at 16–17 days post-infection. (**A**) The frequency of MHV68 DNA-positive splenocytes in C57BL/6J neonatally infected mice was determined by limiting dilution PCR. For this figure, data were compiled from five independent experiments (*N* = 5). Exp 1: M = 2, F = 4; Exp 2: M = 3, F = 2; Exp 3: M = 2, F = 5; Exp 4: M = 6, F = 0; Exp 5: M = 3, F = 3. (**B**) The inverse frequency was converted to %MHV68 DNA-positive cells, displayed for each independent study. Connected symbols represent data from the same study. (**C**) The frequency of *ex vivo* MHV68 reactivation from splenocytes of neonatally infected C57BL/6J mice was determined by limiting dilution assay. For this figure, data were compiled from three independent experiments (*N* = 3). Exp 1: M = 2, F = 4; Exp 2: M = 3, F = 2; Exp 3: M = 6, F = 0. (**D**) The total number of splenocytes per mouse. Data for panel D were compiled from five independent litters (*N* = 5), with distribution of mice as follows: Exp 1: M = 2, F = 4; Exp 2: M = 3, F = 2; Exp 3: M = 2, F = 5; Exp 4: M = 6, F = 1; Exp 5: M = 3, F = 3. Total numbers of mice: mock = 5, male = 16, female = 15. The frequency (**E**) and total number (**F**) of germinal center B cells, gated and defined as in Fig. 2. Data for panels E and F were compiled from four independent litters (*N* = 4), with distribution of mice as follows: Exp 1: M = 2, F = 4; Exp 2: M = 3, F = 2; Exp 3: M = 6, F = 1; Exp 4: M = 3, F = 3. Total numbers of mice: mock = 3, male = 14, female = 10. The frequency (**G**) and total number (**H**) of T follicular helper cells, gated and defined as in Fig. 2. Data for panels G and H were compiled from four independent litters (*N* = 4), with distribution of mice as follows: Exp 1: M = 2, F = 4; Exp 2: M = 3, F = 2; Exp 3: M = 6, F = 1; Exp 4: M = 3, F = 3. Total numbers of mice: mock = 3, male = 14, female = 10. Each symbol represents an individual spleen, and mean and standard error of the mean are shown.

Intranasal inoculation of C57BL/6J neonatal mice with 400 PFU MHV68 failed to reliably establish a chronic latent reservoir in the spleen (Fig. 1D). To determine the extent to which C57BL/6J neonatal mice were resistant to a higher dose MHV68 inoculation, male and female littermates between 8 and 10 days old were inoculated with 4,000 PFU intranasally, and parameters of splenic infection were measured 16–17 days later. Unlike that observed following low-dose inoculation, increased MHV68 inoculum led to the robust establishment of a chronic latent reservoir in the spleen following neonatal infection, as determined by the frequency of MHV68 DNA+ splenocytes and *ex vivo* reactivation (Fig. 7A, B, and C). MHV68 DNA+ splenocytes were reliably identified in the spleens of both male and female neonatally infected mice (Fig. 7A and B). Furthermore, *ex vivo* MHV68 reactivation from splenocytes was observed in both male and female infected neonates, with the frequency of reactivation ~4-fold higher in females (*P* = 0.17, Fig. 7C).

Next, splenomegaly was assessed, as determined by the absolute number of cells per spleen. In contrast to that observed in neonatally infected BALB/c mice, the absolute number of splenocytes was similar between neonatally infected C57BL/6J males, females, and mock-infected controls (Fig. 7D). Both the percentage and absolute number of germinal center B cells were increased in neonatally infected male and female C57BL/6J mice following a higher dose of viral inoculum (Fig. 7E and F, gating strategy as in Fig. 2B). Correspondingly, the percentage and absolute number of T follicular helper cells were also increased in neonatally infected male and female C57BL/6J mice (Fig. 7G and H, gating strategy in Fig. 2E). Thus, while C57BL/6J neonates were resistant to the establishment of systemic infection following low-dose MHV68 inoculation, a higher dose overcame this resistance, as evidenced by the establishment of latent viral reservoir in the spleen, *ex vivo* reactivation, and MHV68-driven germinal center responses.

## DISCUSSION

EBV infection of human adolescents, but not children, results in the development of IM, a risk factor for the later development of cancer and autoimmune disease (2, 3). Consequently, most published MHV68 studies have modeled MHV68 infection in adolescent mice. However, a majority of EBV infections worldwide occur during early childhood, with neonatal immune responses potentially modifying the course of chronic infection and viral pathogenesis (16, 17). Importantly, childhood EBV infection is associated with the development of Burkitt’s lymphoma and childhood Hodgkin’s lymphoma; therefore, there remains a need to improve modeling of gammaherpesvirus infection in pediatric populations (8–10). Using the tractable MHV68 animal model, in this study we show that infection of neonatal BALB/c mice results in robust establishment of splenic latency and *ex vivo* viral reactivation similar to that historically observed following adolescent infection. MHV68 infection of BALB/c neonates induced an increase in the frequency and total number of splenic germinal center B cells and T follicular helper cells, with a majority of the MHV68 latent reservoir housed by germinal center B cells, observations that mirror those following adolescent infection. Interestingly, splenomegaly, a hallmark clinical presentation of EBV and MHV68 infection in adolescents, was only observed in neonatally infected BALB/c males but not females, revealing an intriguing sex-dependent difference in host responses following neonatal MHV68 infection. Finally, C57BL/6J mice were found to be largely resistant to the establishment of systemic MHV68 infection, a resistance that was overcome by increasing the MHV68 inoculum dose.

### Genetic background as a modifier of susceptibility to systemic gammaherpesvirus infection

The current study demonstrates that neonatal infection with 400 PFU of MHV68 failed to reliably establish systemic infection in C57BL/6J mice. Interestingly, in a published study examining the timeline of primary herpesvirus infection of neonates, the oropharyngeal shedding of EBV by mothers or household contacts (as determined by percent of positive samples and EBV copy numbers) was similar for infants remaining uninfected with EBV and infants that acquired EBV infection. These observations suggest that exposure of neonates to EBV+ saliva, as reported by the mothers involved in the study, did not uniformly result in the establishment of EBV infection in neonates (49).

Interestingly, resistance to systemic MHV68 infection has not previously been reported in adolescent mice. In fact, inoculation of C57BL/6J adolescent mice with as little as 40 PFU of MHV68 intranasally reliably establishes systemic infection 16 days later (34). A previous study from the Nice group showed that C57BL/6J neonates demonstrate resistance to mouse rotavirus infection due to increased baseline type III IFN signaling in the gastrointestinal tract (48). While type III IFN can be expressed by multiple cell types, the expression of type III IFN receptors is mostly limited to epithelial cells that are also infected by MHV68 in the lung, at least during acute infection of adolescents (50). However, baseline expression of Mx1 and Mx2, two representative ISGs, was not modified by the genetic background in neonatal lungs (Fig. 5), suggesting that differential baseline IFN signaling, whether driven by type I or III IFN, is unlikely to be responsible for the observed resistance phenotype. Passive immunity mediated by the transplacental transfer of maternal antibodies plays a critical role in protection against neonatal virus infections. Importantly, this was not a factor in the observed study as neonatal infection occurred in pups that were born to MHV68-negative breeders.

Differential genetic background-based susceptibility to neonatal MHV68 infection may reflect differences in T cell responses. Neonatal CD8 T cell responses are unique as they are driven by cytokines and can be independent of TCR ligation (51). The C57BL/6J immune responses are generally skewed toward effector-based immunity, while the BALB/c immune responses are biased toward humoral-based immunity. Thus, it is tempting to speculate that the function of innate-like CD8 T cells may be more robust in C57BL/6J as compared to BALB/c neonates, supporting the observed infection outcomes.

It is also possible that, despite the lack of detectable MHV68 reservoir in the spleen of neonatally infected C57BL/6J mice, the virus persists at very low levels in the upper and/or lower respiratory tract. In support of this hypothesis, treatment of C57BL/6J mice with CpG, a TLR9 ligand, at 30 days post-neonatal infection (400 PFU) increased MHV68 DNA levels and viral reactivation in lungs and spleens; host or viral parameters prior to 30 days post-neonatal infection were not examined (33). It is possible that a subsequent immune-stimulating event, such as acute infection with an unrelated viral or bacterial pathogen, could trigger systemic spread of an initially anatomically restricted gammaherpesvirus reservoir in a neonate. This possibility is supported by published studies demonstrating increased MHV68 reactivation in adolescently infected mice that were subsequently inoculated with Hemophilus influenzae, LCMV (Armstrong), or coxsackie virus (52, 53).

Finally, while the observed genetic background-based difference in the establishment of systemic MHV68 infection has been referred to as resistance, it is possible that the observed phenotypes represent a combination of lower susceptibility and higher resistance. Adolescent C57BL/6J mice are highly susceptible to very low doses of intranasal MHV68 (40 PFU) (34), as compared to neonates in this study. While there are multiple developmental differences between neonates and adolescents, a dramatic and sustained increase in sex hormones (both estrogens and androgens) is a defining feature of adolescence. The role of sex hormones in several acute virus infections is well-established (54–56). In contrast, the proviral role of estrogen has only been shown in the context of Herpes Simplex Virus 1 infection (57). Thus, an important future direction would involve defining the extent to which the increase in sex hormones during adolescence contributes to the increased susceptibility toward systemic gammaherpesvirus infection and subsequent disease.

## MATERIALS AND METHODS

### Animal infections

BALB/cJ and C57BL/6J mice were obtained from The Jackson Laboratory (Bar Harbor, ME) and bred and housed in a specific-pathogen-free barrier facility at the Medical College of Wisconsin (MCW). For infection studies, litters of mice between 8 and 10 days old were inoculated intranasally (5 μL/mouse) with 400 PFU of MHV68 or MHV68.orf73βla (46) diluted in sterile serum-free Dulbecco’s modified Eagle’s medium (Corning, Tewksbury, MA). MHV68 and MHV68.orf73βla viral stocks were prepared and titered on NIH 3T12 cells.

### Limiting dilution assays

The frequency of MHV68 DNA-positive splenocytes and the frequency of *ex vivo* reactivation of MHV68 were determined as previously described (58). Briefly, to determine the frequency of MHV68 DNA-positive splenocytes, splenocytes were pooled from all females or males within each infected litter (2–6 mice/group), and six threefold serial dilutions of splenocytes were subjected to a nested PCR reaction (12 replicates/dilution) using primers against the MHV68 genome (outer forward: 5′-GAGATCTGTACTCAGGCACCTGT-3′; outer reverse: 5′-GGATTTCTTGACAGCTCCCTGT-3′; inner forward: 5′-TGTCAGCTGTTGTTGCTCCT-3′; and inner reverse: 5′-CTCCGTCAGGATAACAACGTCT-3′). To determine the frequency of *ex vivo* reactivation, splenocytes were pooled from all females or males within each infected litter (2–6 mice/group), and eight twofold serial dilutions of splenocytes were plated onto MEF monolayers (24 replicates/dilution). Cytopathic clearing of MEFs was determined after 21 days of culture. To control for preformed virus, four twofold serial dilutions of mechanically disrupted splenocytes were plated on MEFs, and cytopathic effect was determined 14 days post-plating. The proportion of positive events in each dilution (PCR positive or cytopathic effect positive) was plotted against the initial concentration of cells in each dilution. The frequency of positive events (MHV68 DNA-positive or reactivating splenocytes) was determined based on Poisson distribution.

### Persistent lung assays

To measure persistent replication of MHV68, lungs from individual mice were homogenized in 1 mL of DMEM (Corning, Tewksbury, MA, USA) containing 10% fetal bovine serum (FBS) in the presence of sterile 1 mm zirconia beads (catalog number 9835, RPI Research Products, Mount Prospect, IL, USA) using Magnalyzer (Roche). Homogenates (1:10 dilution using 10% fetal bovine serum-supplemented DMEM) were plated onto MEF monolayers (12 replicates/dilution). The proportion of wells exhibiting cytopathic clearing of MEFs was determined after 14 days of culture.

### Flow cytometry

Single cell suspensions of splenocytes were prepared in fluorescence-activated cell sorter (FACS) buffer. A total of 3 × 10^6^ cells were incubated with an optimized concentration of antibody on ice for 30 min. Data were acquired using Celesta flow cytometer (BD Biosciences, Franklin Lakes, NJ, USA) or Cytek Aurora flow cytometer (Cytek Biosciences, Fremont, CA, USA) and analyzed using FlowJo software (BD Biosciences, Franklin Lakes, NJ, USA). The following antibodies were purchased from BioLegend (San Diego, CA, USA): B220-PECy7 (cat. 103222), CD19-FITC (cat. 115506), GL7-APC (cat. 144618), CD95-PE (cat. 152608), CD3-BV421 (cat. 100227), CD4-FITC (cat. 100406), PD1-BV605 (cat. 135220), and CXCR5-PECF594 (cat. 145522).

### qRT-PCR studies

Lungs from individual mice were homogenized in 1 mL of Trizol reagent (Invitrogen) using 1 mm zirconia beads (catalog number 9835, RPI Research Products, Mount Prospect, IL, USA) in the Magnalyzer (Roche). RNA was isolated, DNAse-treated, reverse-transcribed, and analyzed by quantitative reverse transcription-PCR (qRT-PCR) using CFX Connect System (Bio-Rad, Hercules, CA, USA) as previously described (59). No RT reactions were used as controls for genomic DNA contamination. Primers were validated and used within the corresponding linear range. Relative gene expression was quantified by the delta-delta Ct method against *Gapdh* expression. The following primer sequences were used:

Gapdh Forward (5′-TGTGATGGGTGTGAACCACGAGAA-3′); Gapdh Reverse (5′-GAGCCCTTCCACAATGCCAAAGTT-3′); Mx1 Forward (5′-AGCTAGACAGAGCAAACCAAGCCA-3′); Mx1 Reverse (5′-TCCCTGAAGCAGACACAGCTGAAA-3′); Mx2 Forward (5′-AGCAGAGTGACACAAGCGAGAAGA-3′); Mx2 Reverse (5′-AGCCCTTCTGTCCCTGAATCACAA-3′).

### Viral DNA measurement in the lungs

Lungs from individual mice were homogenized in 1 mL of DMEM (Corning, Tewksbury, MA, USA) supplemented with 10% FBS in the presence of sterile 1 mm zirconia beads (catalog number 9835, RPI Research Products, Mount Prospect, IL, USA) using the Magnalyzer (Roche) and digested with proteinase K overnight. DNA was extracted using phenol/chloroform and precipitated overnight in ethanol. DNA was analyzed by quantitative-PCR (q-PCR) using CFX Connect System (Bio-Rad, Hercules, CA). Primers were validated and used within the corresponding linear range. Relative MHV68 DNA was quantified using the delta-delta Ct method against *Gapdh* expression. The following primer sequences were used:

Gapdh Forward (5′-TGTGATGGGTGTGAACCACGAGAA-3′); Gapdh Reverse (5′-GAGCCCTTCCACAATGCCAAAGTT-3′); Orf6 Forward (5′-GTTGCCAGATATCCCTAGGATGA-3′); and Orf6 Reverse (5′-ACCTGGCTGGGTCAAGAGACT-3′).

### ELISA

Enzyme-linked immunosorbent assay (ELISA) to measure serum total IgM, IgG, MHV68-specific IgG and IgM, and anti-double-stranded DNA IgG was performed as previously described (60).

### MHV68 reporter virus studies

Neonatal mice were intranasally inoculated with 400 PFU of MHV68.orf73βla, and spleens were examined at 16–17 days post-infection. Single-cell suspensions of pooled splenocytes were prepared in FACS buffer. A total of 20 × 10^6^ cells were incubated with an optimized concentration of antibody on ice for 30 min. Beta-lactamase activity was detected using LiveBLAzer FRET B/G system according to the manufacturer’s instructions (Thermo-Fisher Scientific). Data were acquired using Cytek Aurora flow cytometer (Cytek Biosciences, Fremont, CA) and analyzed using FlowJo software (BD Biosciences, Franklin Lakes, NJ). The following antibodies were purchased from BioLegend (San Diego, CA) and used in this study: B220-AlexaFluor 647 (cat. 103226), CD19-Spark YG 570 (cat. 115580), GL7-PECy7 (cat. 144620), and CD95-APC/Fire 810 (cat. 152623).

### Statistics

Statistical analyses comparing two groups were performed using the nonparametric Mann-Whitney test. Significance was denoted as *P < 0.05, **P < 0.01, ***P < 0.001, and ****P < 0.0001. Statistical analyses were performed using Prism (GraphPad Software, Inc., San Diego, CA).

## Data Availability

All data associated with the study are available in the article.
